# Candidate genes for domestication and resistance to cold climate according to whole genome sequencing data of Russian cattle and sheep breeds

**DOI:** 10.18699/VJGB-23-56

**Published:** 2023-09

**Authors:** N.S. Yudin, D.M. Larkin

**Affiliations:** Institute of Cytology and Genetics of the Siberian Branch of the Russian Academy of Sciences, Novosibirsk, Russia; Royal Veterinary College, University of London, London, United Kingdom

**Keywords:** signatures of selection; ; ; ; ; ; ;, adaptation, cold, cattle, sheep, local breed, Russia, whole genome sequencing, признаки селекции, адаптация, холод, крупный рогатый скот, овца, местная порода, Россия, полногеномное секвенирование

## Abstract

It is known that different species of animals, when living in the same environmental conditions, can form similar phenotypes. The study of the convergent evolution of several species under the influence of the same environmental factor makes it possible to identify common mechanisms of genetic adaptation. Local cattle and sheep breeds have been formed over thousands of years under the influence of domestication, as well as selection aimed at adaptation to the local environment and meeting human needs. Previously, we identified a number of candidate genes in genome regions potentially selected during domestication and adaptation to the climatic conditions of Russia, in local breeds of cattle and sheep using whole genome genotyping data. However, these data are of low resolution and do not reveal most nucleotide substitutions. The aim of the work was to create, using the whole genome sequencing data, a list of genes associated with domestication, selection and adaptation in Russian cattle and sheep breeds, as well as to identify candidate genes and metabolic pathways for selection for cold adaptation. We used our original data on the search for signatures of selection in the genomes of Russian cattle (Yakut, Kholmogory, Buryat, Wagyu) and sheep (Baikal, Tuva) breeds. We used the HapFLK, DCMS, FST and PBS methods to identify DNA regions with signatures of selection. The number of candidate genes in potentially selective regions was 946 in cattle and 151 in sheep. We showed that the studied Russian cattle and sheep breeds have at least 10 genes in common, apparently involved in the processes of adaptation/selection, including adaptation to a cold climate, including the ASTN2, PM20D1, TMEM176A, and GLIS1 genes. Based on the intersection with the list of selected genes in at least two Arctic/Antarctic mammal species, 20 and 8 genes, have been identified in cattle and sheep, respectively, that are potentially involved in cold adaptation. Among them, the most promising for further research are the ASPH, NCKAP5L, SERPINF1, and SND1 genes. Gene ontology analysis indicated the existence of possible common biochemical pathways for adaptation to cold in domestic and wild mammals associated with cytoskeleton disassembly and apoptosis.

## Abstract (ru)

Известно, что различные виды животных при обитании в одинаковых условиях среды могут сформировать сходные фенотипы. Изучение конвергентной эволюции нескольких видов под действием одного и того же средового фактора позволяет выявить у них общие механизмы генетической адаптации. Местные породы крупного и мелкого рогатого скота формировались на протяжении тысяч лет под воздействием доместикации, а также отбора, направленного на адаптацию к факторам местной среды обитания и удовлетворение потребностей человека. Ранее нами был выявлен ряд генов-кандидатов в участках генома, подвергшихся отбору в ходе доместикации и адаптации к климатическим условиям России, включая низкие зимние температуры, у местных пород крупного рогатого скота (КРС) и овец с использованием данных полногеномного генотипирования. Однако эти данные обладают низким разрешением и не позволяют выявить большинство нуклеотидных замен. Целью работы было создание по данным полногеномного секвенирования списка генов, связанных с адаптацией российских пород КРС и овец, а также идентификация генов-кандидатов и метаболических путей для проведения селекции на адаптацию к холоду. Использованы опубликованные нами данные по поиску следов отбора в геномах российских или разводимых в России пород КРС (якутская, холмогорская, бурятская, вагю) и овец (забайкальская, тувинская). Количество генов-кандидатов в районах, потенциально подвергавшихся селекции, составило 946 у КРС и 151 у овец. Нами показано, что изученные российские породы КРС и овец имеют не менее 10 общих генов под отбором, по-видимому, участвующих в процессах адаптации/селекции, в том числе адаптации к холодному климату, включая гены ASTN2, PM20D1, TMEM176A, GLIS1. На основании пересечения со списком генов, подвергавшихся отбору по крайней мере у двух видов арктических/антарктических млекопитающих, у КРС и овец выявлено 20 и 8 генов соответственно, которые потенциально вовлечены в адаптацию к холоду. Среди них наиболее перспективными для дальней- ших исследований являются ASPH, NCKAP5L, SERPINF1 и SND1. Анализ генных онтологий указывает на суще- ствование возможных общих биохимических путей адаптации к холоду у домашних и диких млекопитающих, связанных с разборкой цитоскелета и апоптозом

## Introduction

The impact of extreme environmental factors can lead to either the extinction of a species or its adaptation to new environmental conditions (Nevo, 2011). It is known that different animal species, inhabiting similar conditions, can develop similar phenotypes using similar biochemical pathways (Storz, 2016). Studying convergent evolution of several species under the influence of the same environmental factor allows for the identification of common genetic adaptation mechanisms (Romashov et al., 2022).

For example, the same non-synonymous mutation in the rhodopsin gene independently arose and was subjected to selection in at least 20 species of fish in response to changes in water light conditions (Hill et al., 2019). The convergent amino acid substitutions also occurred in the prestin gene of whales and bats during the evolution of echolocation (Liu Y. et al., 2010). The non-synonymous substitution His207Arg in the melanocortin 1 receptor gene is associated with light feather coloration in red-footed boobies and ruffs (Lamichhaney et al., 2016). Our recent work on the northernmost cattle, the Yakut cattle from Siberia, revealed the phenomenon of convergent nucleotide evolution among domestic breeds and wild species living in similar harsh conditions and/or exhibiting similar phenotypes. We found the same amino acid substitution in the NRAP protein in Yakut cattle and 16 species of cold-adapted, hibernating or deep-diving mammals, which was absent in all other breeds of cattle and other Bovinae species in the “1000 Bull Genomes” dataset (Buggiotti et al., 2021). According to our data, this amino acid substitution presumably arose 500–800 years ago and is almost fixed in the modern Yakut cattle population.

It is known that the domestication of animals of different species is accompanied by a number of similar morphophysiological and behavioral changes (Belyaev, 1979; Wilkins et al., 2014). For example, one of the typical morphological features of domestication is the disruption of melanin synthesis, as well as a slowing down of melanocyte development, leading to the appearance of white spots on the body, up to the emergence of a uniform white color (Prasolova, Trut, 1993). Such phenotypic parallelism is observed in cattle, horses, pigs, dogs, cats, minks, chickens, pigeons, etc. (Larkin, Yudin, 2016). Indeed, when studying the genomes of populations of domestic animals, strong selection signals have been found in melanin metabolic pathway genes (KIT, KITLG, MITF, PAX3) (Cieslak et al., 2011).

Local breeds of cattle and sheep have been formed over thousands of years under the influence of domestication, as well as natural and artificial selection directed towards adaptation to the factors of the local environment and meeting human needs (Moiseeva et al., 2006; Kantanen et al., 2015). Studying the genomes of local breeds of cattle allows for the identification of genetic mechanisms of adaptation, including to low temperatures of the surrounding environment (Yudin et al., 2021). Earlier, we identified a number of candidate genes in genome regions that were potentially subject to selection during domestication and adaptation to harsh climatic conditions in Russia, in local breeds of cattle (Bos taurus) and sheep (Ovis aries) using data from whole-genome genotyping on standard SNP arrays (Yurchenko et al., 2018, 2019). Based on these results, we also identified 31 common candidate genes related to adaptation to the environment, including cold climate, in animals of the studied breeds (Yudin, Larkin, 2019). For example, the NEB gene, probably associated with heat production through shivering thermogenesis, was identified by us in genome regions subject to positive selection both in native Russian breeds of cattle and sheep, as well as in the genomes of the mammoth, polar bear, and minke whale.

However, whole-genome genotyping data have low resolution and do not allow the detection of most nucleotide substitutions in the genomes of different agricultural animal species. The aim of this study was to create a list of common genes associated with environmental adaptation in Russian breeds of cattle and sheep, as well as to identify promising genetic variants/candidate genes/metabolic pathways for further experiments, marker-assisted and genomic selection aimed at cold adaptation in agricultural animals, using whole-genome sequencing data. Previously, we analyzed selection signatures in the DNA samples from Yakut, Kholmogory, and Buryat cattle using GeneSeek Genomic Profiler High-Density SNP array containing approximately 139,000 SNPs (Yurchenko et al., 2018), and from Baikal and Tuva sheep using Ovine Infinium HD SNP BeadChip (Yurchenko et al., 2019).

## Materials and methods

In the study, we used our own published data on the search for selection signatures using whole-genome sequencing in the genomes of Russian or bred in Russia cattle breeds (Yakut, Kholmogory, Buryat, Wagyu) (Buggiotti et al., 2021; Igoshin et al., 2023) and sheep (Baikal, Tuva) (Sweet-Jones et al., 2021). High-throughput sequencing was performed in paired-end mode (150 bp + 150 bp) on the Illumina platform at Novogene Co., Ltd. (Hong Kong, China) for 20 animals per breed. The average coverage depth was at least 11x for cattle and 15x for sheep.

To identify regions potentially under selection pressure in the genomes of Buryat and Wagyu cattle, we used four complementary methods (Igoshin et al., 2023). The hapFLK method is based on statistics that consider haplotype structure in populations (Fariello et al., 2013). The DCMS method combines five whole-genome statistics: Fisher’s fixation index (FST), haplotype homozygosity (H1), modified haplotype homozygosity (H12), Tajima’s D index (D), and nucleotide diversity index (Pi) (Ma et al., 2015). The FST method identifies genome regions subject to selection by identifying DNA segments with high allelic frequency variability between compared populations (Porto-Neto et al., 2013). The PBS statistic uses pairwise FST values between three populations to quantitatively assess sequence differentiation (Yi et al., 2010). It is considered that genes with high differentiation between sequences may potentially be under positive selection. Candidate gene lists for further analysis of Buryat and Wagyu cattle were compiled by combining lists obtained by different methods. Potential selection regions in the genomes of Yakut and Kholmogory cattle breeds were identified using hapFLK statistics (Buggiotti et al., 2021). For the search for selection signatures in the genomes of Baikal and Tuva sheep, a computational pipeline based on the DCMS method was used (Yurchenko et al., 2019).

Gene identifiers in the Ensembl database were converted into gene symbols using the db2db tool (http://biodbnet.abcc. ncifcrf.gov/db/db2dbRes.php?input=inputType&outputs[]= outputType&idList=value(s)). Intersections between gene lists were analyzed using the Venn program (http://bioinformatics. psb.ugent.be/webtools/Venn/).

Biological functions of the shared genes that were under selection in Russian cattle and sheep breeds, and Arctic/Antarctic mammals were analyzed using the DAVID web tool (Huang et al., 2009). We identified enriched GO terms from the category of biological processes (GOTERM_BP_ALL) associated with four or more genes, compared to the control list of all human genes. We used a significance threshold criterion, characterized by the statistical significance of the observed number of genes with a specific GO term compared to the expected number of genes from the control list, and accepted p < 0.05 as the threshold value.

## Results

The number of candidate genes in regions potentially subjected to selection was 946 for four Russian cattle breeds (List_ Cattle, Suppl. Material 1)1 and 151 for two Russian sheep breeds (List_Sheep, Suppl. Material 2) (see the Table). The difference in the number of candidate genes between species is likely due to differences in the number of breeds included in the analysis, as well as the number of statistical methods used to detect signatures of selection (four statistics for Buryat and Wagyu cattle, one for the other breeds). Analysis of the intersection of the lists showed that 10 genes could potentially have been under selection in both species (see the Figure, Cattle_Sheep list, Suppl. Material 3).

Supplementary Materials are available in the online version of the paper: http://vavilov.elpub.ru/jour/manager/files/Suppl_Yudin_Engl_27_5.pdf


**Table 1.**
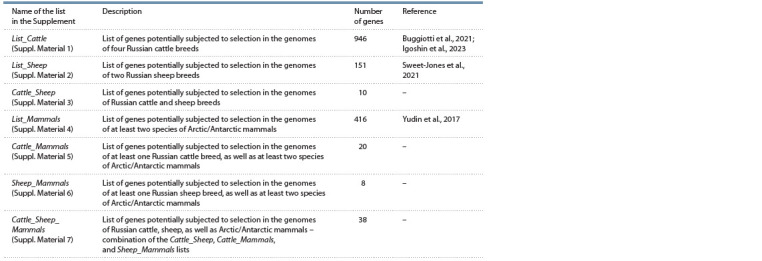
Lists of potentially selected candidate genes

Previously, by intersecting the lists of genes potentially subjected to selection in six Arctic and Antarctic mammal species, we compiled a list of genes that may be involved in cold adaptation (Yudin et al., 2017). The list contained 416 genes that were likely under selection in at least two mammal species (List_Mammals, Suppl. Material 4). To identify common genes that may be associated with adaptation to cold climate in Russian cattle and sheep breeds, we compared the lists of List_Mammals, List_Cattle, and List_Sheep. As a result, we found 20 (Cattle_Mammals, Suppl. Material 5) and 8 (Sheep_Mammals, Suppl. Material 6) genes that were potentially under selection in at least two wild mammal species adapted to cold climate as well as in cattle and sheep, respectively (see the Figure).

To test the hypothesis that these lists were enriched in functional categories of genes related to cold adaptation, we performed gene ontology (GO) analysis on a list of 38 genes obtained by merging the Cattle_Sheep, Cattle_Mammals, and Sheep_Mammals lists (Cattle_Sheep_Mammals, Suppl. Material 7). As a result, we found significant enrichment in eight GO terms that were associated with 4 or more genes (Suppl. Material 8).

## Discussion

Our study aimed to identify common candidate genes in the genomes of domestic cattle and sheep breeds in Russia that may have undergone selection and played a role in adaptation to extreme climates, as well as to identify promising genetic variants/candidate genes/metabolic pathways for further cold adaptation research. We identified a total of 10 genes that potentially could have been under selection simultaneously in both Russian cattle and sheep breeds (Cattle_Sheep list, see Suppl. Material 3). These genes were likely subjected to selection during domestication and/or subsequent selection for economically important traits, as well as during adaptation to cold climates.

According to the theory of D.K. Belyaev, numerous morphophysiological transformations in domestic animals are caused by destabilizing selection for the absence of aggressive behavior towards humans (Belyaev, 1979). Indeed, we have previously shown that a list of 1262 common genes that underwent selection in Russian cattle and sheep breeds through whole-genome genotyping was enriched in genes predominantly expressed in the brain (Yudin, Larkin, 2019). Several common genes identified in our study (see Suppl. Material 3) are expressed in nervous tissue and are involved in normal neuron function. For example, the protein astrotactin 2 (ASTN2) modulates synaptic activity in neurons by regulating the expression of synaptic proteins in post-migratory neurons via endocytosis (Behesti et al., 2018). Genetic variants in the ASTN2 gene are associated with Alzheimer’s disease (Wang et al., 2015), schizophrenia (Autism Spectrum Disorders Working Group of The Psychiatric Genomics Consortium, 2017), autism (Lionel et al., 2014), and other psychiatric disorders. The gene encoding the protein containing a peptidase domain M20 1 (PM20D1) is associated with Alzheimer’s disease (Sanchez-Mut et al., 2018) and Parkinson’s disease (Rudakou et al., 2021). The transmembrane protein TMEM176A gene is associated with schizophrenia (Kos et al., 2017).

At the same time, deletion in the ASTN2 gene results in a reversal of normal orientation of hair follicles in adult mice (parallel to “from head to tail”) to the opposite direction (parallel to “from tail to head”) (Chang et al., 2015). In humans, the ASTN2 gene is associated with the level of triglycerides in the blood (Jiao et al., 2015) and the development of obesity (Burt et al., 2021). Signatures of selection in this gene have been found in ethnic groups of southern Ethiopia, who have lived in high-altitude conditions for over a thousand years (Scheinfeldt et al., 2012). Interestingly, adaptive introgression of a large number of ancient Neanderthal alleles has been identified in the ASTN2 gene in the population of South Asia (Racimo et al., 2017). The biochemical pathway of PM20D1 modulates the accumulation of brown fat and thus participates in the process of heat production through non-shivering thermogenesis (Gao et al., 2018). The pro-adipogenic factor GLIS1 may play a critical role in the differentiation of mesodermal cells during fetal development and affect fat distribution in the tail of sheep (Luo et al., 2021). SNP polymorphism in the NSG1 gene is associated with the fat content in milk of Holstein cows (Lee et al., 2016).

We investigated promising genetic variants and candidate genes for cold adaptation by intersecting the lists List_Cattle and List_Sheep with the List_Mammals of 416 genes that were positively selected in at least two species of Arctic/Antarctic mammals (Yudin et al., 2017). When all three lists of common genes and genetic variants were intersected, none were found (see the Figure), but we identified 20 and 8 genes (see Suppl. Materials 5 and 6), respectively, that were potentially subjected to selection during adaptation to the climate of both Arctic mammals and cattle or sheep, respectively.

**Fig. 1.**
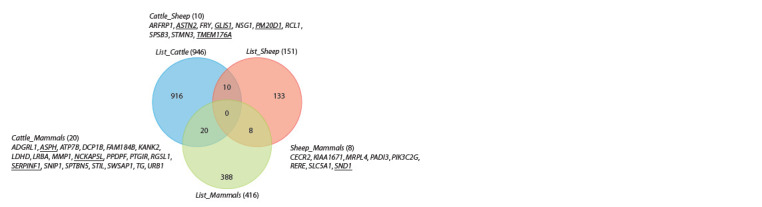
Venn diagram showing overlaps between lists of genes potentially subjected to selection in the genomes of Russian cattle (List_Cattle) and sheep (List_Sheep) breeds, and at least two species of Arctic/Antarctic mammals (List_Mammals). The number of genes in each list is indicated in parentheses. The most promising cold adaptation candidate genes (based on their biological role) are underlined.

Thus, genetic variants in the ASPH gene, which encodes a protein that regulates the process of excitation–contraction in muscles, are associated with heat stroke and malignant hyperthermia in humans (Endo et al., 2022). According to whole-genome association analysis, single nucleotide polymorphisms in this gene are associated with intramuscular fat distribution in beef cattle (Ramayo-Caldas et al., 2014). Genetic variants in the NCKAP5L (Chen et al., 2013) and SERPINF1 (Böhm et al., 2012) genes are associated with the development of obesity in humans. The human gene FAM184B is associated with body composition and fatty acid profile (Yuan et al., 2021). The protein product of the PADI3 gene controls hair shape on the human scalp (Liu F. et al., 2018). The mRNA expression of the gene of the protein containing the staphylococcal nuclease domain 1 (SND1) in the New Zealand alpine stick insect significantly increases in response to cold exposure (Dunning et al., 2013). In mammals, SND1 plays an important role in regulating lipid metabolism through the activation of the SREBP2 protein (Navarro-Imaz et al., 2020).

The gene ontology terms identified by the DAVID program when analyzing the list of potentially selected genes in Russian cattle, sheep, as well as Arctic/Antarctic mammals (Cattle_ Sheep_Mammals list) can be divided into three groups: (1) terms related to the disassembly of cell parts and protein complexes (“disassembly of cell components”, “disassembly of protein complex”, “disassembly of macromolecular complex”, etc.); (2) terms related to DNA disintegration (“hydrolysis of phosphodiester bonds in nucleic acids”); (3) uninformative terms of the top hierarchy describing general biological processes (“biological process occurring at the level of a multicellular organism”) (see Suppl. Material 8).

Enrichment of gene ontology terms related to the disassembly of cell parts, proteins, and DNA may be the result of natural selection for genes encoding cytoskeletal proteins and/or participating in programmed cell death (apoptosis). Studies on hibernating mammals have shown that their cells respond to low temperatures by disassembling the cytoskeleton and delaying apoptosis (Van Breukelen, Martin, 2002). It is believed that cytoskeletal disassembly may be the cause of protein synthesis suppression in mammalian cells during cold stress (Al-Fageeh, Smales, 2006). Hypothermia causes disassembly of microtubules by activating p38 MAP kinase in human retinal cells (Thanuja et al., 2021). In in vivo and in vitro experiments, it has been shown that microtubules in peripheral axons of Xenopus are sensitive to cold, and their density varies depending on the season (Alvarez, Fadić, 1992). It has been shown that cold stress induces apoptosis of neurons in the hippocampus of mice (Xu et al., 2019).

In our study, the term “disassembly of cellular components” was associated with seven genes (see Suppl. Material 8). Among them, the gene SPTBN5 encodes one of the spectrin family proteins, which are common components of the cytoskeleton, interacting with elements of the cell scaffold and plasma membrane, providing proper localization of major membrane proteins, signal transmission into the cell, and other processes (Beijer, Züchner, 2022). The protein NCKAP5L, interacting with the protein CDK5RAP2, regulates microtubule stability in HeLa cells (Mori et al., 2015). The protein stathmin-3, encoded by the STMN3 gene, regulates the rapid reorganization of the cytoskeleton in response to environmental factors by affecting the balance of microtubule assembly and disassembly (Nair et al., 2014). With the term “hydrolysis of phosphodiester bonds of nucleic acids”, genes RCL1, CECR2, SND1, and DCP1B were associated (see Suppl. Material 8). It has been shown that the SND1 protein suppresses apoptosis in hepatocellular carcinoma cells by interacting with the long non-coding RNA UCA1 (Cui et al., 2018). The CECR2 protein is localized in the DNA condensation regions of apoptotic human liver cells and interacts with the chromatin-associated protein TAFII30 (Liu L. et al., 2002).

## Conclusion

Thus, using whole-genome sequencing data, we have shown that the studied Russian cattle and sheep breeds have at least 10 common genes, presumably involved in adaptation/ selection processes, including adaptation to cold climate, such as ASTN2, PM20D1, TMEM176A, GLIS1. Based on the overlap with the list of genes subjected to selection in at least two species of Arctic/Antarctic mammals, 20 and 8 genes potentially involved in adaptation to cold were identified in cattle and sheep, respectively. Among them, the most promising for further research are the genes ASPH, NCKAP5L, SERPINF1, and SND1. Gene ontology analysis indicates the existence of possible common biochemical pathways for adaptation to cold in domestic and wild mammals, related to cytoskeleton disassembly and apoptosis.

## Conflict of interest

The authors declare no conflict of interest.
